# The Mechanism of Damage to the Midgut by Low Concentration of *Bacillus thuringiensis* in the Silkworm, *Bombyx mori*

**DOI:** 10.3390/insects15120911

**Published:** 2024-11-21

**Authors:** Hongbin Zou, Haoyi Gu, Jialu Cheng, Chao Tian, Qilong Shu, Peilin Peng, Bing Li

**Affiliations:** 1 School of Life Sciences, Suzhou Medical College, Soochow University, Suzhou 215006, China; 20224021002@stu.suda.edu.cn (H.Z.); 20234021006@stu.suda.edu.cn (H.G.); 20224221054@stu.suda.edu.cn (J.C.); ctian@stu.suda.edu.cn (C.T.); 18985575232@163.com (Q.S.); pengpl0321@163.com (P.P.); 2 Sericulture Institute, Soochow University, Suzhou 215006, China

**Keywords:** *Bombyx mori*, *Bacillus thuringiensis*, midgut, apoptosis, transcriptome analysis

## Abstract

*Bacillus thuringiensis* (Bt) has been extensively applied in agricultural pest management, posing a notable ecological risk to beneficial insects like *Bombyx mori* (silkworms). However, the toxicological mechanisms of Bt at low concentrations on silkworms remain largely unexplored. In this study, we determined the *LC*_50_ (96 h) of Bt for fifth-instar silkworm larvae to be 0.08 × 10^−3^ mg/L. Exposure to a sub-lethal concentration of Bt (1/2 *LC*_50_) led to significant reductions in body weight, pupal size, and the weights of both the whole cocoon and cocoon shell. Histopathological and ultrastructural examinations revealed that Bt exposure caused severe damage to the microvilli and epidermal cells of the midgut. Transcriptome sequencing of the midgut identified 290 differentially expressed genes (DEGs), with these genes predominantly involved in metabolic processes and apoptotic pathways. Notably, apoptosis-related genes such as *Apaf-1* and *Caspase-3* were upregulated by 5.08-fold and 1.27-fold, respectively. Further validation through TUNEL assays and Western blotting analysis confirmed a significant activation of apoptotic signaling. These findings suggested that low concentrations of Bt could trigger apoptotic pathways in the midgut of silkworm larvae, providing valuable insights into the toxicological evaluation of Bt at sub-lethal doses in insect species.

## Simple Summary

*Bacillus thuringiensis* (Bt), as a kind of microbial insecticide, kills harmful insects while also harming beneficial insects such as *Bombyx mori* Linnaeus, 1758 (Lepidoptera: Bombycidae). However, the toxicological mechanisms of low-concentration Bt on silkworm have not been extensively studied. Our study found that low concentrations of Bt caused poisoning in *Bombyx mori*, possibly by activating the apoptotic pathway. We screened out apoptosis genes in *Bombyx mori* and analyzed their expression in midgut tissue. These results provide a basis for further investigating the toxicological mechanisms of low concentrations of Bt against insects.

## 1. Introduction

*Bacuillus thuringiensis* (Bt) is a Gram-positive microorganism that produces a range of proteins during both sporulation and vegetative growth phases [1,2]. Once solubilized and proteolytically activated in the insect midgut, these transform into potent toxins that compromise the midgut’s integrity, ultimately causing the insect’s death [3,4,5]. Bacuillus thuringiensis is not only easy to cultivate but also demonstrates low persistence and significant insecticidal effects [6,7]. As a result, Bt has been applied as a biological insecticide against lepidopteran, coleopteran, dipteran, and other pests for over five decades [8,9]. However, the extensive use of Bt has raised concerns regarding its chronic toxicity in non-target organisms due to environmental residues. Studies have shown that exposure to low concentrations of Bt can adversely affect the larval development of various insect species, including *Aedes aegypti (Linnaeus, 1762)*, *Leptinotarsa decemlineata (Say, 1824)*, *Helicoverpa armigera (Hübner, 1808)*, and *Sesamia nonagrioides (Lefèbvre, 1827)* [10,11,12,13,14]. Despite these findings, the molecular mechanisms underlying midgut injury caused by low-dose Bt exposure in insects remain poorly understood.

*Bombyx mori* Linnaeus, 1758 (Lepidoptera: Bombycidae), a lepidopteran insect, holds significant economic and scientific value [15,16]. It has become highly sensitive to external environmental factors after more than 5000 years of selective breeding [17,18]. Many Bt crops transformed with genes encoding various *Bacillus thuringiensis* (Bt), such as Bt rice, which have shown resistance to one or more lepidopteran pests of rice in the field, such as yellow stem borer (*Scirpophaga incertulas*) [19]. The widespread cultivation of Bt crops, such as Bt rice and maize, has resulted in the dispersion of Bt pollen onto mulberry leaves through wind, impacting silkworms that rely on these leaves for sustenance, and this exposure has led to a marked reduction in silk production [20,21,22].

Apoptosis is one of the signaling pathways of programmed cell death [23], which plays a vital role in biological growth and development, as well as cell proliferation and cell differentiation [24]. Apoptosis is characterized by morphological changes and biochemical events such as cell contraction, vacuolization, chromatin condensation, DNA fragmentation, exteriorization of phosphatidylserine, cell phagocytosis, and activation of caspases such as caspase 3, 8, and 9. Caspase activation can occur by ligating membrane-binding receptors or by mitochondrial depolarization and release of cytochrome c and APAF-1 [25].

The early study found that Bt could induce the vacuolation of midgut cells in *Bombyx mori* [26]. Vacuolation and nuclear condensation of midgut cells in other insects were subsequently observed [27,28]. Swelling and vacuolation of mitochondria have also been found in other tissues such as the malpighian tubule [29]. However, it has not been suggested that these phenomena are closely related to apoptosis signals, and morphological changes have only been observed through light and electron microscopy, without involving specific biochemical experiments. Our study firstly identified apoptosis as a primary mechanism underlying midgut damage caused by low concentrations of Bt. Then, we further verified the apoptotic signal by morphology and molecular chemistry. These findings opened up a new avenue of research into the effects of low-dose insecticides on non-target organisms.

## 2. Materials and Methods

### 2.1. Insects and Chemicals

The silkworm larvae (Huakang 3 strain) were purchased from Rugao Sericulture Station (Nantong Jiangsu, China), fed with fresh mulberry leaves (Yu-711 strain) three times in our laboratory, the Sericulture Institute of Soochow University, under standard conditions (25 ± 1 °C, 12:12 h light/dark photoperiod, 75 ± 5% relative humidity). Bt (8000 IU/μL) was purchased from Beijing Green Agricultural Science and Technology Group Co., Ltd. (Beijing, China).

### 2.2. Toxicity Evaluation and Low Concentration of Bt Treatment

*Bacuillus thuringiensis* (8000 IU/μL) is dissolved in sterilized water and diluted to different working solutions (0.01 × 10^−3^, 0.04 × 10^−3^, 0.05 × 10^−3^, 0.1 × 10^−3^, and 0.2 × 10^−3^ mg/L). Mulberry leaves were soaked in different solutions for 1 min and air-dried naturally for about 30 min. Three liters of working solution were used to soak the mulberry leaves. Silkworm larvae at the third day of the fifth instar were randomly selected for Bt treatment until cocooning. The treatment group was fed Bt-treated mulberry leaves, and the control group was fed sterilized-water-treated leaves. Thirty insects per replicate were used for survival statistics. Three insects per replicate were chosen for dissection. Thirty insects per replicate were collected to measure the body weight and cocooning rate. The pupa and cocoon were photographed and observed. Mortality was defined as the index of poisonous silkworms, which was recorded at 96 h. The *LC*_50_ (lethal concentration 50) value and 95% confidence intervals were calculated using standard probit analysis [30].

### 2.3. Sample Collection and Extraction of Total RNA

The midgut (MG) of silkworms was dissected on ice at 96 h following Bt (0.04 × 10^−3^ mg/L) exposure, immediately cleaned in pre-cooled 1× PBS buffer (Sangon Biotechnology Co., Ltd., Shanghai, China), and dried with sterilized filter paper. Three midgut tissues were randomly selected and placed into 1.5 mL RNase-free tubes, frozen instantly in liquid nitrogen, and finally stored at −80 °C for the next experiments.

Frozen tissues were thawed, ground into powder with a sterilized mortar, and 50 mg of each sample was weighed out. Total RNA from the midgut tissues was extracted using RNAiso Plus (Takara, Dalian, China), and impurities were removed with trichloromethane, isopropanol, and 75% ethanol. The quality of RNA was detected with 1% agarose gel electrophoresis and NanoDrop-2000 (Thermo Fisher Scientific, Boston, MA, USA).

### 2.4. Transcriptome Sequencing and Identification of Differentially Expressed Genes (DEGs)

High High-quality RNA was used as material for the construction of cDNA libraries, and 150-base paired-end reads were generated on the DNBSEQ™ platform (BGI). Sequencing data were filtered with SOAPnuke, and paired-end clean reads were mapped and spliced to the silkworm reference genome using HISAT2 [31,32]. Then, the transcriptional location information was identified and revised by STRINGTIE [33,34]. Finally, HTSEQ was adopted to calculate the number of reads and the level of gene expression [35]. DESeq2 was used to screen DEGs between two groups with the criteria of adjusted q-value < 0.05 and | Log_2_FoldChange | > 0.5 [35]. Gene Ontology (GO) and Kyoto Encyclopedia of Genes and Genomes (KEGG) annotation and enrichment pathway analysis of DEGs were performed to assign putative functional annotations.

### 2.5. Quantitative Real-Time PCR (qRT-PCR) Analysis

The Reverse Transcriptase M-MLV reagent kit was purchased from Sangon Biotechnology Co., Ltd. (Shanghai, China) for synthesizing cDNA. The primers (Table S1) for qRT-PCR were designed on NCBI (Primer designing tool (nih.gov)) and synthesized by Sangon Biotechnology Co., Ltd. (Shanghai, China). Each 10 μL qRT-PCR reaction mixture contained 25 ng cDNA, 0.4 μL of each primer (10 μM), 5 μL TB Green *Premix Ex Taq* II (2×) (Takara, Dalian, China), and 4.1 μL sterilized water. Quantitative real-time PCR was performed on a CFX Opus Real-time PCR System (Bio-Rad, USA). The tubes were incubated at 95 °C for 30 s, followed by 40 cycles of the thermal program (5 s at 95 °C, 30 s at 60 °C). *Actin*3 was selected as an internal reference gene [36]. Three biological and technical replicates were performed for each gene. The relative transcription levels of target genes were measured using the 2^−ΔΔCt^ method [37] and visualized using GraphPad Prism 8.0.1.

### 2.6. Immunohistochemistry (IHC) and Transmission Electron Microscopy (TEM)

The fresh midgut tissues were fixed in 4% paraformaldehyde, embedded in paraffin, and then sliced into 5 μm sections and fixed on glass slides. The histopathological sections were examined blindly with the Nikon DS-U3 (Nikon, Tokyo, Japan) imaging system after hematoxylin-eosin staining.

The fresh midgut tissues were fixed in 4% glutaraldehyde, then dark fixed at 4 °C for 2 h, washed with 1 × PBS buffer three times, and fixed with 1% osmium tetroxide for 1 h, dehydrated with a graded series of ethanol (75%, 85%, 95%, and 100%), stained with 0.5% uranyl acetate in water overnight, and after that, coated with Epon 812 and stained with lead citrate and uranium dioxide acetate. Finally, 100 nm sections were obtained with the Leica UC6 (Leica, Berlin, Germany) and examined under HT7700 transmission electron microscopy (Hitachi, Tokyo, Japan).

### 2.7. Apoptosis Detection in Frozen Sections of Midgut

The fresh midgut tissues were cleaned with 1 × PBS buffer, then frozen sections were prepared according to the manufacturer’s instructions using a previously described protocol [38]; subsequently, they were stained with TUNEL Kit (chromogenic method) (Beyotime, Shanghai, China), and 2-(4-Amidinophenyl)-6-indolecarbamidine dihydrochloride (DAPI) (Solarbio, Beijing, China) was used for staining cell nuclei, and the Nikon DS-U3 (Nikon, Japan) imaging system was used for detecting apoptotic signals.

### 2.8. Western Blot Assay

The midgut samples were lysed in lysis buffer (Beyotime, Shanghai, China) with 1mM phenylmethylsulfonylfluoride (Beyotime, Shanghai, China). The lysed tissues were centrifuged at 12,000 rpm at 4 °C for 10 min, then the supernatant was collected and the protein concentration was determined by the BCA method [39]; finally, 60 μg of total protein was used for the Western blot assay. The prepared samples were separated using 10% SDS-PAGE and transferred to PVDF membranes. The membrane was incubated with primary antibodies at 4 °C overnight after being blocked with 5% BSA in TBST at 25 °C for 2 h. The primary antibodies used were β-tubulin at a dilution of 1:1000 (CST, Boston, MA, USA) and Caspase-3 at a dilution of 1:500 (GenScript, Nanjing, China). The primary antibody-binding was visualized with a horseradish peroxidase (HRP)-conjugated goat anti-rabbit IgG (CST, Boston, MA, USA) at a dilution of 1:1000 at 25 °C for 1 h. All results of the Western blot were revealed by using the Chemiluminescent HRP Substrate Kit (Bio-Rad, Hercule, CA, USA) and image analysis software (ImageJ 2.1.4.7, Bethesda, MD, USA).

### 2.9. Statistical Analysis

Statistical analysis was performed with SPSS 19.0 (SPSS, USA) using Student’s *T*-test and one-way analysis of variance (ANOVA). The level of significance with *p* < 0.05 (*) and *p* < 0.01 (**) was considered with statistical significance. All quantitative data were presented as mean ± standard deviation (SD). All figures were drawn with GraphPad Prism 8 (GraphPad Software, San Diego, CA, USA).

## 3. Results

### 3.1. Toxicity of Bt Product Against Silkworms

**Figure 1:**
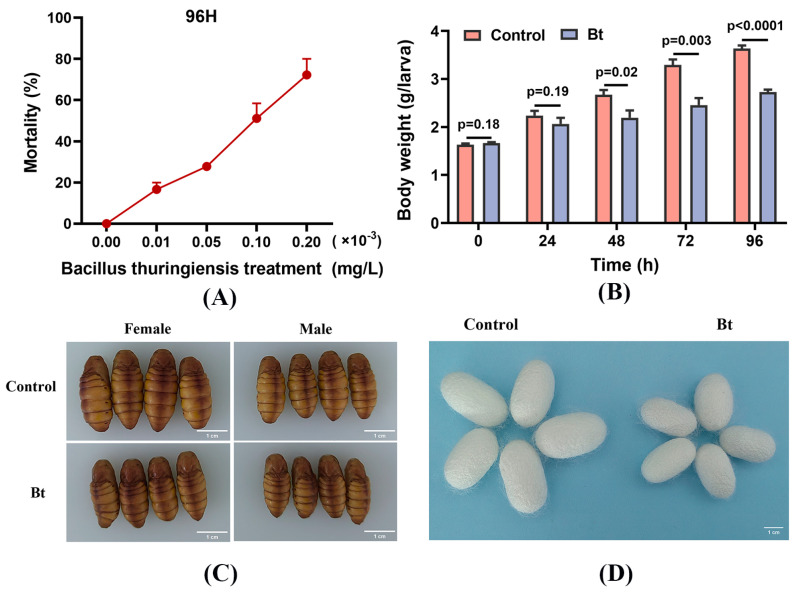
The toxicity and effect of Bt treatment on the growth of silkworms. (**A**) The toxicity curve of different concentrations of Bt treatment to silkworms at 96 h. (**B**) The effect of Bt treatment on larval body weight of silkworm. (**C**) The effect of Bt treatment on pupae. (**D**) The effect of Bt treatment on cocoons. Scale bar of 1 cm.

Silkworm larvae were fed mulberry leaves treated with varying concentrations of Bt, and mortality was recorded after 96 h (Table S2). The mortality curve indicated that Bt exerted cumulative damage on silkworms (Figure 1A). Based on a toxic regression equation (y = 0.425x + 0.9383), the *LC*_50_ (96 h) for fifth-instar silkworm larvae was determined to be 0.08 × 10^−3^ mg/L. Following Bt exposure for 96 h, larval body weight decreased by 24.9% (Figure 1B). Additionally, Bt significantly impacted the size of both pupae and cocoons, leading to a marked reduction in whole cocoon and cocoon shell weight (Figure 1C,D). However, the proportion of the cocoon shell to the total cocoon weight remained unaffected by Bt treatment (Table 1).

### 3.2. Histopathological and Ultrastructural Analysis of the Midgut

**Figure 2:**
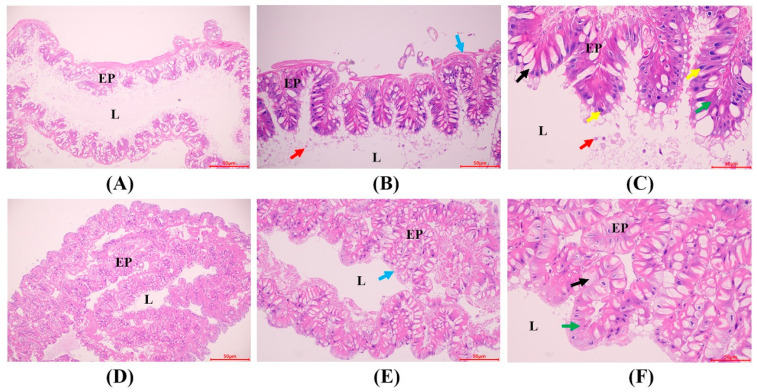
Histopathological changes in the midgut of silkworm larvae at 96 h after Bt exposure. (**A**–**C**) Represent the Bt treatment group. (**D**–**F**) represent the control group. Columnar cells (green arrow), goblet cells (black arrow), basement membrane (blue arrow), epithelium (EP), lumen (L), cells fell off (red arrow), and chromatin condensation (yellow arrow). Scale bar of 50 μm.

Histopathological analysis was performed to assess Bt-induced damage to the midgut. In the Bt-treated group, the midgut cell layer was thinner, with numerous nuclei displaced into the lumen, and the microvilli were detached from the underlying structure, and chromatin was contracted (Figure 2A–C). In contrast, the midgut of the control group displayed well-preserved epithelial cells, with tightly organized goblet and columnar cells and an intact, clearly defined basement membrane (Figure 2D–F).

**Figure 3:**
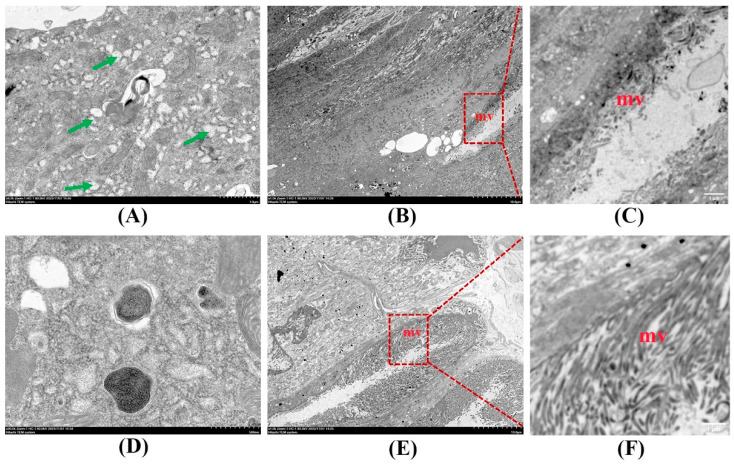
Ultrastructural of silkworm larvae exposed to Bt treatment for 96 h. (**A**,**B**) The ultrastructural observation in the Bt treatment group, and (**C**) is the enlarged view of (**B**) with a red dotted box. (**D**,**E**) The ultrastructural observation in the control group, and (**F**) is the enlarged view of (**E**) with a red dotted box. Microvilli (Mv), vacuoles (green arrow).

Transmission electron microscopy (TEM) further revealed ultrastructural changes in midgut cells. In the Bt-treated group, numerous vacuoles were observed (Figure 3A). The microvilli had sloughed off from goblet cells, leading to significant loss (Figure 3B,C). In the control group, there were few vacuoles (Figure 3D), while the microvilli appeared dense and uniformly distributed (Figure 3E,F). These ultrastructural findings aligned with the histopathological observations.

### 3.3. Transcriptome Sequencing Analysis and Gene Identification in Silkworm Midguts

To investigate the molecular mechanisms underlying the silkworm response to Bt, transcriptome sequencing was conducted on midgut samples from both control and Bt-treated groups. This analysis generated over 265 million raw reads, with approximately 44 million clean reads obtained from each library, except for the B1 group. The Q20 and Q30 scores exceeded 97.5% and 92.1%, respectively, ensuring high data quality (Table S3). More than 85.6% of the filtered reads were successfully mapped to the reference genome, with 71.0% to 74.4% of these reads uniquely aligned (Table S3).

**Figure 4:**
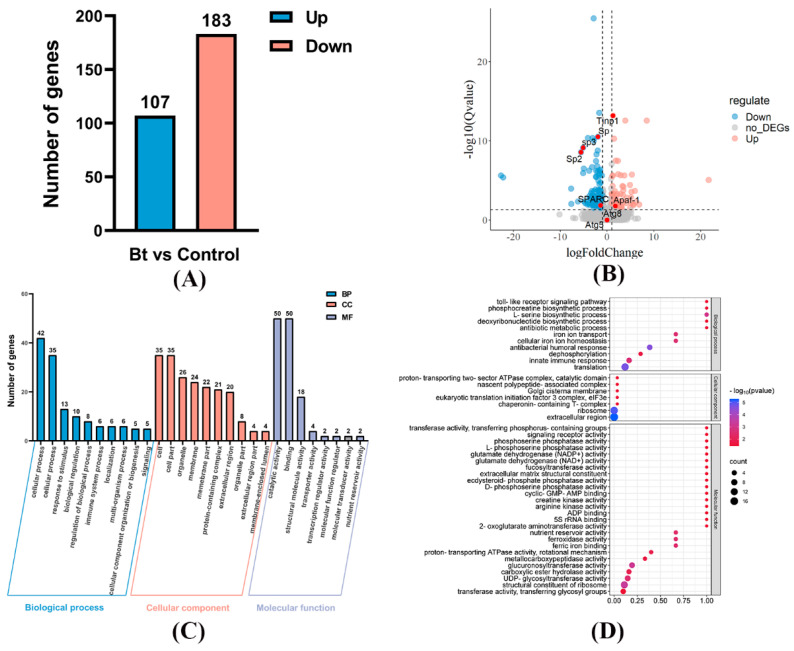
Transcriptomic analysis in the midgut of silkworm larvae after exposure to low concentrations of Bt. (**A**) Quantities of up- and down-regulated DEGs. (**B**) Volcano map for transcriptomic analysis between Bt-treated and control groups. Blue and pink dots represent significant up- and down-regulated DEGs, respectively. The red dots are symbolic genes. (**C**) Summary of Gene Ontology (GO) classification of DEGs. (**D**) Dot plot of the GO enrichment analysis.

In total, 21,454 transcripts were detected, including 12,509 novel transcripts. Furthermore, 290 DEGs were identified between the control and Bt treatment groups, with a |Log_2_FoldChange| greater than 0.5 and a q-value below 0.05. Among these DEGs, 107 genes were significantly upregulated, while 183 were significantly downregulated (Figure 4A,B). A detailed breakdown of the DEGs is provided in Table S4.

### 3.4. Gene Functional Annotation, GO and KEGG Pathway Enrichment Analysis, and Key Gene Identification

Functional categorization was performed using the Gene Ontology (GO) database to investigate the functional roles of the DEGs. The most significantly enriched GO subcategories included “cellular process” (77), “binding” (50), and “catalytic activity” (50) (Figure 4C). Furthermore, GO enrichment analysis revealed that genes associated with “translation”, “ribosome”, and “antibiotic metabolic process” were most affected by Bt exposure (Figure 4D).

**Figure 5:**
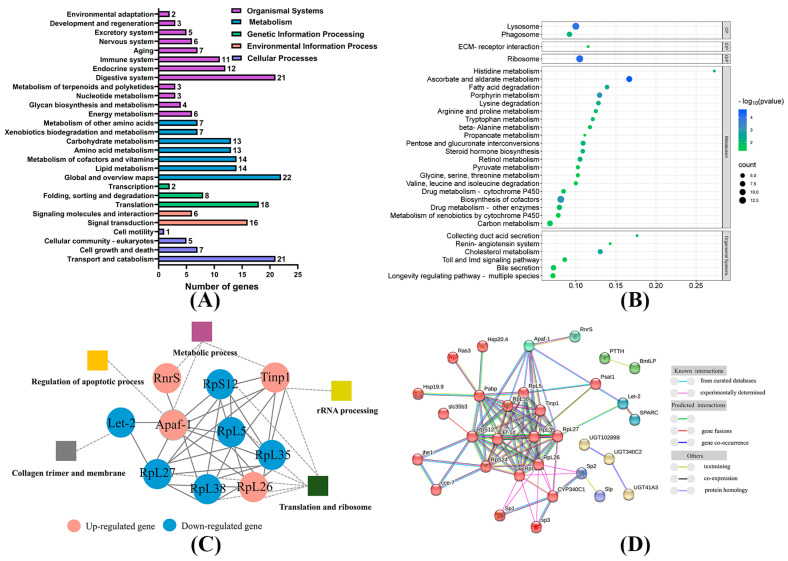
Transcriptomic analysis in the midgut of silkworm larvae after exposure to low concentrations of Bt. (**A**) Summary of KEGG classification of DEGs after Bt exposure. (**B**) Dot plot of the KEGG enrichment analysis. The horizontal axis shows the gene proportion, and the vertical axis shows the enriched pathway name. Genetic Information Processing (GIP), Cellular Processes (CP), Environmental Information Processing (EIP). The color scale represents different thresholds of *p*-value (*p* < 0.05), and the size of the dots represents the quantities of genes corresponding to each pathway. (**C**) The interaction diagram of apoptosis-related proteins. Different colored nodes represent different MCL clusters (find natural clusters based on the stochastic flow). (**D**) The interaction diagram of proteins related to apoptotic, metabolic processes; rRNA processing; collagen trimers and membranes; and translation and ribosome pathways. Gray dotted lines indicate GO pathways. Network nodes and edges indicate proteins and protein-protein associations, respectively.

To gain deeper insights into the biological changes induced by Bt treatment in the silkworm midgut, KEGG pathway analysis was applied to all DEGs. The pathways were classified into five main categories, with the top three being “cellular process”, “organismal systems”, and “metabolism” (Figure 5A). Additionally, KEGG enrichment analysis identified 30 significantly enriched pathways (*p* < 0.05), with the majority of DEGs being concentrated in metabolic pathways, followed by organismal systems pathways (Figure 5B).

A protein–protein interaction (PPI) network comprising 49 proteins was constructed using STRING and Cytoscape, which revealed seven distinct clusters (Figure 5C). Notably, these proteins were predominantly associated with processes such as apoptosis, metabolism, collagen trimer formation, and membrane structure (Figure 5D).

### 3.5. Effect of Bt on the Apoptosis Levels in Silkworm Midguts

**Figure 6:**
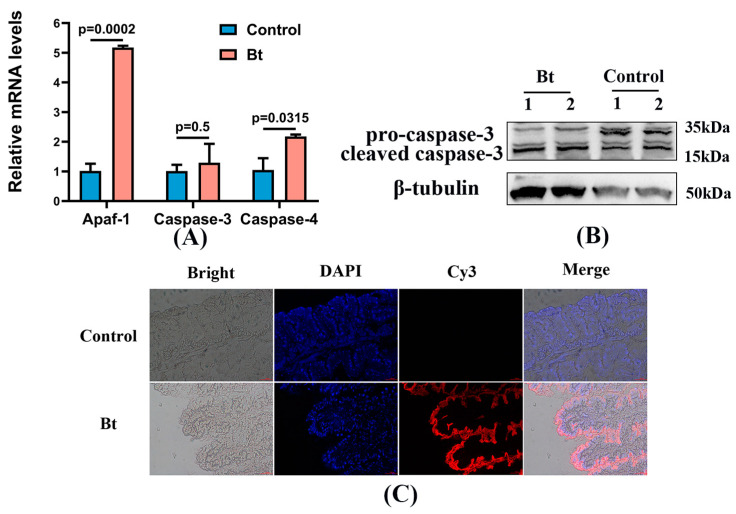
Low concentrations of Bt exposure promote apoptosis in the midguts of silkworms. (**A**) Transcript levels of apoptosis-related *Apaf*-1, *Caspase*-3, and *Caspase*-4. (**B**) Western blotting analysis of Caspase-3, numbers 1 and 2, represents two biological duplications. (**C**) TUNEL assay in frozen sections of the midguts. Cell nuclei are stained with DAPI (blue); apoptotic signal is represented by Cy3 (red). The results are expressed as mean ± SD, and the significance is determined by *t*-test and *p*-value, and *p*-value < 0.05 was statistically different. Scale bar of 100 μm.

To assess the activation of apoptotic pathways after Bt treatment exposure, the transcription levels of three apoptosis-related genes were measured using qRT-PCR. The results revealed significant upregulation of *Apaf-1*, *Caspase-3*, and *Caspase-4*, with increases of 5.08-fold, 1.27-fold, and 1.38-fold, respectively (Figure 6A). Additionally, protein levels of Caspase-3 showed a 1.33-fold increase (*p* < 0.01), which aligned with the observed mRNA expression levels (Figure 6B). Apoptosis was further evaluated using TUNEL assays, which demonstrated widespread apoptotic signals in the midgut tissue of Bt-exposed silkworms compared to the control group (Figure 6C).

## 4. Discussion

In the evaluation of the toxic side effects of pesticides, most studies have focused on assessing the sublethal effects of long-term exposure to pesticides through food consumption in insects [40]. Low concentrations of insecticides, including biopesticides, can negatively influence various aspects of a pest’s life cycle, including growth, development, and reproductive capacity [41,42]. In our study, we determined the *LC*_50_ (96 h) of Bt for fifth-instar silkworm larvae to be 0.08 × 10^−3^ mg/L. Prolonged exposure to this low concentration of Bt (96 h) significantly reduced larval body weight, as well as pupal and cocoon weights, while causing notable damage to the midgut. Furthermore, transcriptomic analysis, along with gene and protein validation, revealed the activation of apoptosis pathways. These findings offered a new perspective on the mechanisms of action of low-concentration Bt in non-target organisms.

LC50 (Lethal concentration 50) represents the dose or inhaled concentration of a substance that causes death in 50% of a tested population, making it a critical metric for evaluating toxicity [43]. Our findings showed that a significantly lower concentration of Bt at 0.08 × 10^−3^ mg/L, was sufficient to achieve the same lethal effect in silkworms, highlighting their heightened sensitivity to Bt compared to other pesticides, such as indoxacarb, imidacloprid, and thiamethoxam, reported as 1.08 × 10^2^, 1.92, 0.66, and 1.66 mg/L, respectively [43,44]. This underscored the importance of studying Bt’s impact on silkworms. Moreover, our research revealed that critical biological traits, including body weight, cocoon weight, and cocoon layer ratio, were severely compromised after exposure to low concentrations of Bt. In conclusion, even low concentrations of Bt could cause substantial harm to silkworms.

The midgut epithelium is the primary target of Bt, making it the focus for assessing the harmful effects of Bt on silkworms [5]. Previous studies have documented midgut damage in other pests exposed to Bt, reporting adverse effects such as cellular hypertrophy, microvilli damage, vacuolization, mitochondrial swelling, and an increase in lysosomes [45,46,47]. Our research similarly revealed significant alterations in the midgut epithelium of silkworms following exposure to low concentrations of Bt, which is in accordance with these earlier findings. These results suggest that midgut damage from Bt exposure is a common phenomenon, likely affecting the growth and development of silkworms.

Transcriptomic sequencing analysis identified 290 DEGs, with GO and KEGG pathway enrichment analyses revealing that the majority of these DEGs were significantly associated with metabolic processes, including lipid, amino acid, and carbohydrate metabolism. Our findings were consistent with previous studies on silkworms exposed to chlorfenapyr [48]. Similarly, recent RNA-seq research on *Helicoverpa zea (Lepidoptera: Noctuidae)* has demonstrated significant alterations in lipid and glucose metabolism following Bt exposure [49]. Moreover, the PPI network indicated a connection between metabolic processes and apoptotic pathways. As with other insecticides, disruption of metabolic processes can damage silkworm midguts and initiate apoptosis [50,51,52]. Collectively, these results suggest that the toxic mechanism of low-concentration Bt may be intricately linked to metabolic pathways.

In this study, the expression of pro-apoptotic genes (*Apaf-1* and *Caspase-4*) was elevated, while Caspase-3, a key apoptosis inducer [53], showed a significant increase at the protein level. In addition, strong apoptotic signals were detected in TUNEL-stained frozen midgut sections. These findings clearly demonstrated that apoptotic pathways were activated in the silkworm midgut following exposure to low concentrations of Bt. Apoptosis can occur via multiple pathways, including the extrinsic and intrinsic pathways, the latter involving the endoplasmic reticulum, mitochondria, and lysosomes [54]. Overall, we hypothesized that low concentrations of Bt induced midgut damage by activating the apoptotic pathway. Further investigation is needed to determine the specific type of apoptosis induced by Bt in the silkworm midgut. Our research showed that low concentrations of Bt could lead to damage to the silkworm by inducing apoptosis in the midguts. These results not only provided a direct basis for the reasonable use of mulberry fields but also provided a reference for the safety evaluation of Bt in long-term environmental exposure.

## Figures and Tables

**Table 1:** Effect of low concentration of Bt on cocoon quality.

Goup Name	Cocoon Weight (g)	Cocoon Shell Weight (g)	Ratio of Cocoon Shell
Control	1.75 ± 0.03	0.38 ± 0.02	0.22 ± 0.01
Bt	1.12 ± 0.03 ***	0.20 ± 0.03 ***	0.18 ± 0.02

The results were shown as mean ± SEM. SPSS 19.0 was used for one-way ANOVA (*** *p* ≤ 0.001).

## Data Availability

Data are contained within the article.

## Supplementary Materials

The following supporting information can be downloaded at https://www.mdpi.com/article/10.3390/insects15120911/s1, Table S1: Primer sequences for quantitative real-time PCR; Table S2: Death number of silkworms larvae after exposure to low concentration of Bt for 96 h; Table S3: Summary of the transcriptome sequencing data; Table S4: DEGs between the control and Bt treatment group in the midguts of silkworms.
