# Supplementary material for: The Mechanism of Damage to the Midgut by Low Concentration of Bacillus thuringiensis in the Silkworm, Bombyx mori
Source: Insects. 2024 Nov 21;15(12):911. doi: 10.3390/insects15120911 (PMC11677582; doi:10.3390/insects15120911)
Supplement: Supplementary file 1 [file insects-15-00911-s001.zip › insects-3290925-supplementary.pdf]

insects MDPI

1. Specific primers for apoptosis-related genes for quantitative real-time PCR

Table S1. Primer sequences for quantitative real-time PCR.

Primer name	Primer Sequence (5'-3')	NCBI Gene ID
*BmActin*3-F	CGGCTACTCGTTCACTACC	NM_001126254.1
*BmActin*3-R	CCGTCGGGAAGTTCGTAAG	
BmCaspase-3-F	AGTTTCGGTCATCTGCTTTAC	NM_001043832
BmCaspase-3-R	CATTCGGACTTCCTCTTCTTA	
BmCaspase-4-F	TAATACAGGCATGCAGGGGC	NM_001257006
BmCaspase-4-R	CCATGATCCGTCGACTTCGT	
BmApaf-1-F	TATGCTGCGTCCCCTG	317108134
BmApaf-1-R	GTGCCATTATCTCGTTTTG	

2. Death number of silkworm larvae after Bt treatment

Table S2. Death number of silkworms larvae after exposure to low concentration of Bt for 96 h.

Concentration（$\times 10^{-3}$mg/L）	Mortality (number)		
	group 1	group 2	group 3
0.00	0	0	0
0.01	4	7	4
0.05	8	9	8
0.10	11	17	18
0.20	18	21	26

3. summary of data information for RNA-seq

Table S3. Summary of the transcriptome sequencing data.

Sample	Total Raw Reads (M)	Total Clean Reads (M)	Total Clean Bases (Gb)	Clean Reads Q20 (%)	Clean Reads Q30 (%)	Clean Reads Ratio (%)	Total Mapping (%)	Uniquely Mapping(%)
B1	40.96	40	6	97.6	92.28	97.64	86.70	74.43
B2	45.44	44.41	6.66	97.62	92.36	97.74	86.80	74.36
B3	45.44	44.41	6.66	97.62	92.35	97.73	85.64	73.14
C1	45.44	44.33	6.65	97.64	92.45	97.57	88.15	73.17
C2	45.44	44.1	6.62	97.56	92.16	97.06	16.62	71.02
C3	45.44	44.37	6.66	97.55	92.17	97.66	86.43	71.64

Table S4. DEGs between the control and Bt treatment group in the midguts of silkworms.

Gene ID	Gene Symbol	$\log_2$ (Bt / Control)	q-value (Bt / Control)
100101150	RpS24	0.896529921	0.031111203
100101209	LOC100101209	-0.652140461	0.034070185
100134928	LOC100134928	-1.42570618	0.000000427
100134929	LOC100134929	3.861215426	0.000000000
100134931	Sp1	-6.219256887	0.004471024
100146102	Slp	-3.775863931	0.000962822
100174976	LOC100174976	-1.268504208	0.048067671
100272181	Bm122	3.288376714	0.000051322
100301505	LOC100301505	3.417085397	0.000714789
100313492	Ugt3	-3.190384893	0.000000204
100328605	SCRB4	1.187822347	0.000050383
100379394	CPR79	-3.823286547	0.000000594
100379427	CPR42	-2.25713841	0.000000005
100379461	CPR5	-1.671847624	0.002455456
100500754	UGT10286	-2.373938243	0.000003521
100500755	UGT10287A	-2.420582455	0.011157497
100500757	UGT10289B	-2.566361123	0.009437418

Gene ID	Gene Symbol	$\log_2$ (Bt / Control)	q-value (Bt / Control)
100500761	cce-7	-1.180033035	0.031111203
100529085	Apaf-1	1.774604222	0.016603604
100529205	LOC100529205	-1.962763734	0.031049516
100653512	BmILP	-1.443826441	0.047932271
100862800	sp3	-5.092207428	0.000000001
100862809	UGT340C1	1.23523183	0.011134622
100862810	UGT340C2	-1.392992382	0.026193892
100862816	UGT41A3	-2.080514105	0.012012476
100873153	RnrS	1.659519528	0.000231327
101735307	LOC101735307	-1.024130991	0.000675243
101735319	LOC101735319	0.601072386	0.020974978
101735354	LOC101735354	-1.230769391	0.007300209
101735388	LOC101735388	3.467482815	0.000174291
101735418	LOC101735418	-2.852577178	0.000000000
101735439	LOC101735439	-1.631851143	0.042164394
101735472	LOC101735472	-5.099680036	0.000001137
101735502	LOC101735502	1.254328095	0.011310255
101735597	LOC101735597	1.099873397	0.042780642
101735616	LOC101735616	-1.240521131	0.002494224
101735656	LOC101735656	-0.68271718	0.018841489
101735775	LOC101735775	-3.022314889	0.012662825
101735876	LOC101735876	-1.265271322	0.019263641
101735895	LOC101735895	-1.335608702	0.000018180
101735904	LOC101735904	-4.904597691	0.004445506
101736151	LOC101736151	-1.313678314	0.038147552
101736168	LOC101736168	6.23742138	0.004549455
101736335	LOC101736335	2.037660798	0.010067502
101736365	LOC101736365	5.125215629	0.006839745
101736498	LOC101736498	2.3257647	0.000000033
101736567	LOC101736567	2.180171557	0.000522461
101736620	LOC101736620	-1.36058393	0.002783058
101736655	LOC101736655	2.537693128	0.0277168
101736769	LOC101736769	-1.289665654	0.005209465
101736859	LOC101736859	-3.215766926	0.000205599
101736886	LOC101736886	-1.894439533	0.020583535
101736985	LOC101736985	-1.467590267	0.000962822
101737052	LOC101737052	-2.696372511	0.002382887
101737199	LOC101737199	-2.132820167	0.018579732
101737249	LOC101737249	-2.891715395	0.003908811
101737295	LOC101737295	-4.20029215	0.011134622
101737534	LOC101737534	1.265921801	0.01413336
101737541	LOC101737541	-0.871771754	0.011724819
101737626	LOC101737626	-1.502346159	0.013626041
101737662	LOC101737662	3.449025293	0.000058702
101737697	LOC101737697	-1.612165961	0.000028885
101737711	LOC101737711	-1.169930721	0.047001228
101738141	LOC101738141	-0.844125781	0.020811546
101738261	LOC101738261	-1.690213165	0.000000000
101738353	LOC101738353	-1.203059619	0.041624149
101738432	LOC101738432	-1.38523914	0.000836314
101738588	LOC101738588	-3.039731296	0.010067502
101738597	LOC101738597	-0.911254606	0.047424961
101738769	LOC101738769	4.188548796	0.001575536
101739037	LOC101739037	-1.369392608	0.010508862
101739059	LOC101739059	-2.810101747	0.000852077
101739068	LOC101739068	-0.647964363	0.034791508
101739125	LOC101739125	5.133908772	0.001319752
101739289	LOC101739289	-1.984586373	0.000000639
101739290	LOC101739290	-1.264640731	0.020811546

Gene ID	Gene Symbol	log_2 (Bt / Control)	q-value (Bt / Control)
101739424	LOC101739424	1.068943725	0.012510404
101739502	LOC101739502	2.281217214	0.01884915
101739536	LOC101739536	-3.229928625	0.000946504
101739571	LOC101739571	-1.601116633	0.00454181
101739681	LOC101739681	-2.732713734	0.000962822
101739881	LOC101739881	-1.174671735	0.00777004
101739958	LOC101739958	-2.315091201	0.009437418
101739963	LOC101739963	-1.711223541	0.000551354
101740092	LOC101740092	-3.634054849	0.012606887
101740201	LOC101740201	-2.332133087	0.000000639
101740215	LOC101740215	-0.800597508	0.034070185
101740224	LOC101740224	-2.634140113	0.013547764
101740277	LOC101740277	-1.288498185	0.000246889
101740388	LOC101740388	-2.308200251	0.002382887
101740395	LOC101740395	0.990887887	0.024306463
101740414	LOC101740414	-3.000909339	0.00793453
101740531	LOC101740531	2.932255241	0.000002220
101740533	LOC101740533	-1.097328905	0.007477069
101740553	LOC101740553	1.70597279	0.02857349
101740657	LOC101740657	1.32527959	0.001467968
101740671	LOC101740671	1.872480329	0.000000032
101740697	LOC101740697	4.943546445	0.028379653
101740764	LOC101740764	4.089445122	0.007084269
101740802	LOC101740802	-1.881244651	0.000174291
101740826	LOC101740826	2.302488698	0.008125681
101740845	LOC101740845	-1.685460197	0.000264833
101740958	LOC101740958	0.756428943	0.029802263
101741250	LOC101741250	-0.902712884	0.030641988
101741385	LOC101741385	-2.825135371	0.000000000
101741415	LOC101741415	-1.679491516	0.028184253
101741498	LOC101741498	-1.402533331	0.026516671
101741533	LOC101741533	1.01206413	0.010357887
101741571	LOC101741571	5.931381788	0.000209116
101741585	LOC101741585	-0.791309721	0.031369447
101741616	LOC101741616	0.996089867	0.009767958
101741798	LOC101741798	-1.640177917	0.003087519
101741801	LOC101741801	1.097524084	0.000570639
101741863	LOC101741863	1.675639869	0.00357151
101742139	LOC101742139	0.631227219	0.043297872
101742160	LOC101742160	-1.39738083	0.000215927
101742191	LOC101742191	4.896437032	0.000036380
101742197	LOC101742197	1.60208589	0.031369447
101742274	LOC101742274	-7.698720962	0.000104932
101742279	LOC101742279	-3.939293504	0.006839745
101742342	LOC101742342	1.32492241	0.000425700
101742360	LOC101742360	-0.818697331	0.020038544
101742398	LOC101742398	1.680397831	0.008125681
101742412	LOC101742412	0.934151777	0.000425700
101742493	CYP340C1	1.883364961	0.026953543
101742524	LOC101742524	-2.314757595	0.001009299
101742613	LOC101742613	-2.863620062	0.005845003
101742685	LOC101742685	2.824775498	0.003956725
101742689	LOC101742689	-4.110127914	0.000000000
101742724	LOC101742724	-0.866015474	0.04955222
101742839	LOC101742839	1.059796126	0.000687465
101742843	LOC101742843	8.468428091	0.000000000
101742914	LOC101742914	-0.837020926	0.03985584
101742998	LOC101742998	-1.286245861	0.019263641
101743002	LOC101743002	-2.098057642	0.000000154

Gene ID	Gene Symbol	log_2 (Bt / Control)	q-value (Bt / Control)
101743090	LOC101743090	-0.806336197	0.010943226
101743244	LOC101743244	1.279716562	0.045757036
101743302	LOC101743302	-1.26609385	0.022150751
101743385	LOC101743385	-1.158392981	0.014314459
101743457	LOC101743457	-1.527202177	0.00489292
101743755	LOC101743755	-2.376962728	0.000003277
101743840	LOC101743840	-0.647663399	0.024378971
101743861	LOC101743861	-1.267406009	0.000570639
101744133	LOC101744133	-0.707385969	0.042436758
101744250	LOC101744250	-1.343713348	0.031267141
101744283	LOC101744283	2.390368178	0.000001648
101744336	LOC101744336	-1.217634236	0.011771374
101744348	LOC101744348	1.303181657	0.037983636
101744373	LOC101744373	-0.626954693	0.020811546
101744459	LOC101744459	-3.422637666	0.000428199
101744461	LOC101744461	-1.167928005	0.011245269
101744477	LOC101744477	1.694393092	0.025094515
101744522	LOC101744522	3.088649702	0.023990991
101744534	LOC101744534	1.546515299	0.030576397
101744557	LOC101744557	-1.514166151	0.027483268
101744560	LOC101744560	6.846630416	0.010943226
101744811	LOC101744811	-2.828077896	0.029802263
101744873	LOC101744873	-1.405459603	0.027483268
101745132	LOC101745132	5.193955341	0.000264833
101745140	LOC101745140	0.841163318	0.030416997
101745174	LOC101745174	-2.429109612	0.000139333
101745215	LOC101745215	-3.072536684	0.012061233
101745224	LOC101745224	5.305014838	0.000001850
101745242	LOC101745242	1.022142092	0.042780642
101745252	LOC101745252	-0.8807128	0.038125617
101745363	LOC101745363	-2.763503673	0.000229314
101745415	LOC101745415	0.672674437	0.026801776
101745433	LOC101745433	-1.116060761	0.000002296
101745483	LOC101745483	1.723654629	0.029084834
101745599	LOC101745599	-1.078426922	0.045344142
101745618	LOC101745618	1.477353236	0.000000000
101745694	LOC101745694	1.592904904	0.029802263
101745703	LOC101745703	-1.426939669	0.014661429
101745847	LOC101745847	-1.075840429	0.002336126
101745858	LOC101745858	-1.996322152	0.000000002
101746062	LOC101746062	-0.815179992	0.000871709
101746159	LOC101746159	2.870136469	0.000522461
101746287	LOC101746287	-2.841991138	0.013547764
101746307	LOC101746307	0.763393817	0.035139122
101746362	LOC101746362	-1.732401028	0.000002385
101746390	LOC101746390	-0.755575834	0.031727099
101746462	LOC101746462	-1.632456676	0.000142056
101746662	LOC101746662	3.703721307	0.000002379
101746715	LOC101746715	-1.169889802	0.041454807
101746745	LOC101746745	-3.566217651	0.001853287
101746755	LOC101746755	0.794082347	0.001631354
101746805	LOC101746805	-1.950686925	0.043297872
101746814	LOC101746814	-1.548731007	0.013705371
101746879	LOC101746879	1.502039553	0.004445506
101746969	LOC101746969	-0.570990485	0.038035717
101747054	LOC101747054	-2.571734231	0.003087519
101747092	LOC101747092	0.923308959	0.028426178
101747102	LOC101747102	3.477266369	0.005857907
101747217	LOC101747217	-1.142503097	0.013547764

Gene ID	Gene Symbol	$\log_2$ (Bt / Control)	q-value (Bt / Control)
105841453	LOC105841453	2.472128975	0.017401483
105841539	LOC105841539	-1.695228933	0.00777004
105841586	LOC105841586	-2.502247672	0.006912821
105841933	LOC105841933	-4.956602392	0.000000319
105842100	LOC105842100	-7.624514441	0.009325234
105842201	LOC105842201	1.609164183	0.022123765
105842301	LOC105842301	1.015478932	0.000536298
105842367	LOC105842367	3.112832096	0.020077297
105842437	LOC105842437	2.480845103	0.017401483
105842445	LOC105842445	-1.835288282	0.000010525
105842703	LOC105842703	-0.901112269	0.042753792
105842927	LOC105842927	-1.919221537	0.033902651
119628335	LOC119628335	-1.440212076	0.000520197
119628365	LOC119628365	21.68909995	0.000008501
119628385	LOC119628385	-1.49632636	0.000001330
119628541	LOC119628541	-22.66593467	0.000002381
119628717	LOC119628717	-0.906104228	0.010357887
119628878	LOC119628878	1.325702186	0.024438129
119629177	LOC119629177	-0.830021532	0.000967457
119629384	LOC119629384	2.002928388	0.000792946
119629403	LOC119629403	5.608262011	0.012308301
119629690	LOC119629690	1.099022679	0.015423408
119629863	LOC119629863	1.745325149	0.008703547
119630034	LOC119630034	1.55143551	0.026516671
119630225	LOC119630225	-1.679812115	0.047156524
119630326	LOC119630326	2.891682729	0.029560115
119630562	LOC119630562	-1.332684612	0.011134622
119630849	LOC119630849	-2.357971363	0.000000034
119630860	LOC119630860	-22.20840321	0.000004184
119630891	LOC119630891	-1.52503713	0.000246889
119630946	LOC119630946	-1.912949372	0.006486702
119631031	LOC119631031	5.581677835	0.010321193
119631051	LOC119631051	-1.413156734	0.031049516
119631101	LOC119631101	-1.657553365	0.032549513
692393	Ef-1d	0.865772071	0.02266331
692444	LOC692444	1.797928241	0.002110845
692447	Sp	-1.962641848	0.000000000
692452	Lsp-t	-3.808914244	0.002797721
692487	Hsp20.1	-2.063126728	0.00811699
692488	Hsp23.7	-1.769203122	0.000169114
692508	Cb10	-1.625942975	0.000000558
692519	Ras3	-0.582240441	0.042243639
692533	Hsp19.9	-1.651073167	0.010357887
692550	CPR46	-1.463724624	0.001073731
692557	RpL5	0.987772837	0.000522461
692563	Apn1	-0.928920666	0.006351921
692579	jhe1	-1.411236924	0.027483268
692591	Hsp20.4	-3.092557794	0.000000000
692595	E75	-1.248664382	0.013660995
692625	Let-2	-1.323001108	0.011157497
692630	CTL11	-2.78412616	0.006221147
692632	LOC692632	-1.5581656	0.015352654
692702	RpL26	1.13072334	0.000002815
692703	RpL27	0.968502987	0.001986026
692706	RpL35	0.846359156	0.000101087
692711	RpL39	1.021481299	0.007477069
692721	RpS19	0.779130771	0.049431128
692743	LOC692743	-1.385350201	0.041226422
692767	PTTH	1.866002185	0.044393331

Gene ID	Gene Symbol	log_2 (Bt / Control)	q-value (Bt / Control)
692797	LOC692797	0.65063558	0.003160027
692826	LOC692826	-1.222264018	0.027834319
692839	slc35b3	1.181216132	0.003590774
692889	LOC692889	-1.990210783	0.039919654
692924	AK	-1.377105448	0.01399766
692936	LOC692936	0.641300058	0.008125681
692939	LOC692939	-0.78013176	0.000246889
692953	LOC692953	0.87979422	0.009041256
692964	Psat1	-1.203293123	0.027237502
692972	RpL7Ae	1.232185172	0.01399766
693008	Tinp1	1.253913476	0.000000000
693028	CECB1	-2.48954434	0.011315209
693045	Lp-c23	-4.637957502	0.007702828
693065	RpL29	0.918277455	0.002783058
693081	LOC693081	-1.244084411	0.002498078
693118	RpL38	0.919954959	0.019352107
724046	RpL41	1.204157506	0.000002379
732856	RpS12	0.886830875	0.013705371
732899	LOC732899	0.822751208	0.000006237
732916	LOC732916	-1.476986897	0.019320611
732947	CCE-un1	-1.439870641	0.03985584
732949	LOC732949	-1.425043092	0.02734215
732958	SPARC	-1.415738339	0.013626041
732967	LOC732967	-1.454282918	0.030416997
732982	LOC732982	-0.778118222	0.017795218
732987	LOC732987	-0.903615967	0.022434679
732990	LOC732990	-0.727290232	0.013626041
733005	Sp2	-5.588980865	0.000000003
733016	LOC733016	-1.221525359	0.023844181
733061	LOC733061	-0.83331293	0.000132996
733062	LOC733062	-0.769905295	0.02266331
733140	LOC733140	-1.928142231	0.013778312
767621	Nuc	-2.210219744	0.007135851
778454	RpL36A	0.988238399	0.000000086
778506	LOC778506	-0.935222424	0.000425700
778529	Pabp	0.493831074	0.040213127
791084	LOC791084	0.73537409	0.014944043
